# Giant Ulcerative Dermatofibroma

**DOI:** 10.1155/2013/254787

**Published:** 2013-11-25

**Authors:** Turgut Karlidag, Erol Keles, Israfil Orhan, Mehmet Erkan Kaplama, Bengu Cobanoglu

**Affiliations:** ^1^Department of Otorhinolaryngology, Firat University Medical Faculty, 23100 Elazig, Turkey; ^2^Department of Otorhinolaryngology, Elazig Medical Park Hospital, 23100 Elazig, Turkey; ^3^Department of Pathology, Firat University Medical Faculty, 23100 Elazig, Turkey

## Abstract

Dermatofibroma is a slowly growing common benign cutaneous tumor characterized by hard papules and nodules. The rarely seen erosions and ulcerations may cause difficulties in the diagnosis. Dermatofibrosarcoma protuberans, which is clinically and histopathologically of malignant character, displays difficulties in the diagnosis since it has similarities with basal cell carcinoma, epidermoid carcinoma, and sarcomas. Head and neck involvement is very rare. In this study, a giant dermatofibroma case, which is histopathologically, ulcerative dermatofibroma, the biggest lesion of the head and neck region and seen rarely in the literature that has characteristics similar to dermatofibrosarcoma protuberans, has been presented.

## 1. Introduction

Dermatofibromas are lesions of the fibrous tissue which are painless, solitary, round, hard, well limited, and slowly growing and generally involve the skin [1–3]. Histopathologically, they contain fibroblasts, collagen, and histiocytes [3]. They may be nodular, red, brown, or blue-black [2, 3]. They may be pink or flesh-colored in light-skinned patients. They consist of nodular, collagen, and fibroblastic cells. These benign tumors may be confused with melanomas or soft tissue sarcomas [3]. The rarely observed erosions and ulcerations may lead to mistakes in the clinical diagnosis. Ulcerated dermatofibroma is a recently defined rare dermatofibroma variant, in which destruction can be observed in the epidermis and the superficial dermis [4]. The classical type is a popular lesion which is red, brown, hard, dome-shaped, being slightly raised from the skin, and varying in size, from a few millimeters to 2 cm. It is generally localized in the lower extremities of adult women. The involvement of the head and neck region is very rare [5]. The diagnosis is made by histopathological evaluation of the biopsy obtained from the lesion. The treatment is excisional biopsy [2]. Recurrence is rare in cases that are removed by intact surgical margins [2, 3].

In this study, a giant dermatofibroma case in the head and neck region, which has not been previously defined in the literature, has been presented.

## 2. Case Report

**Figure 1:**
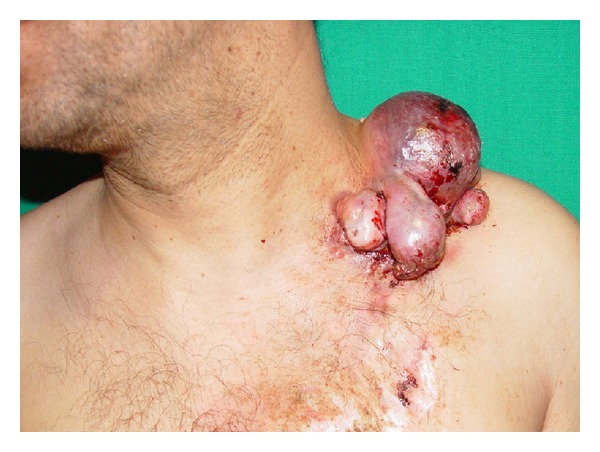
Preoperative image of the mass.

**Figure 2:**
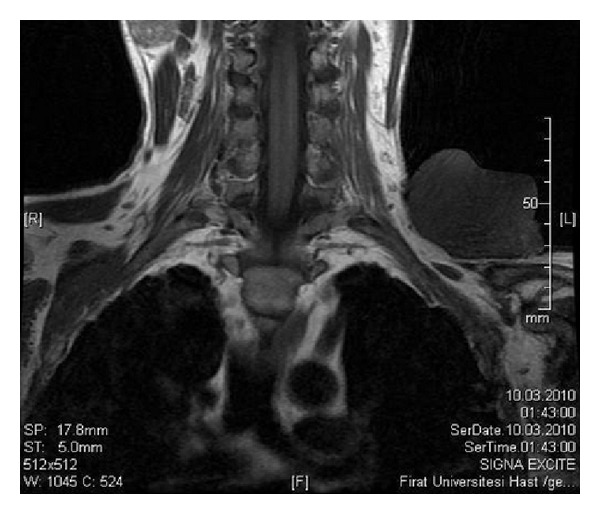
MR image of the mass.

**Figure 3:**
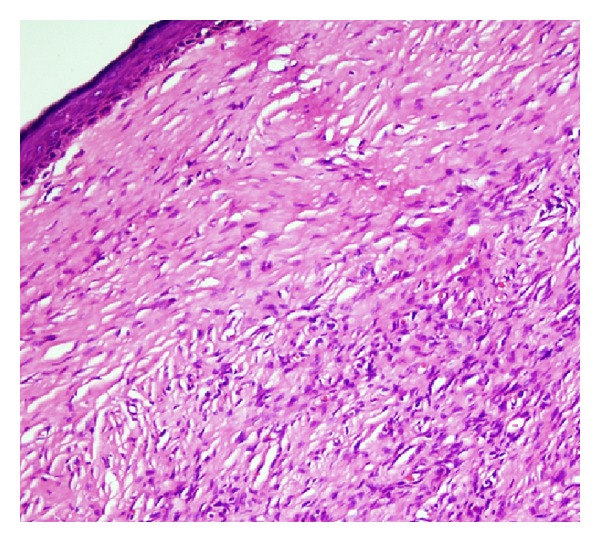
In the pathology preparate, spindle-shaped fibroblasts and bundles of short crossing myofibroblasts are observed (HE 200).

A thirty-six-year-old male patient presented with a mass in the left supraclavicular region, which had been gradually growing in the last year. On his examination, a painless mass approximately 10 × 8 cm in size was observed, which was hard and fixed in the left supra-clavicular region (Figure 1). By magnetic resonance imaging of the neck, a mass of 10 × 8 cm size, which was regularly demarcated and had no invasion to the surrounding muscle and bone structures, was determined (Figure 2). Incisional biopsy of the mass was performed and the mass was diagnosed as dermatofibroma according to the histopathological evaluation. In the operation, it was observed that the mass involved the cutaneous and the subcutaneous tissues, but not the muscle. By leaving a surgical margin, a wide excision was performed on the mass. The skin defect formed following surgery was closed with a posterior scapula flap. The postoperative pathology result was reported as dermatofibroma (Figure 3). In the one-year follow-p of the case, there was no observation of recurrence or complication.

## 3. Discussion

Dermatofibrosarcoma protuberans is a rare tumor of the skin, which comprises 1% of soft tissue sarcomas and less than 0.1% of all malignancies. It is a malignant mesenchymal tumor originating from the dermis layer of the skin. Its differential diagnosis from dermatofibroma is difficult, both clinically and histopathologically [6]. 

In the histopathology of dermatofibroma, spindle-shaped fibroblasts, nodular dermal proliferation with myofibroblasts sequenced as short crossing bundles, foamy cytoplasmic histiocytes, and thick hyaline collagen bundles in the periphery are observed. Furthermore, an increase in the number of blood vessels, erythrocyte extravasations or perivascular infiltration of hemosiderin, lymphocyte and plasma cells, and mitosis can be observed. Generally, there is hyperplasia, union in the rete ends and hyperpigmentation in the basal layer of the epidermis. In immune-histochemical investigations, there are a positive reaction with Factor XIIIa antibody and a negative reaction with CD34 antibody [5]. In 1994, LeBoit and Barr collected dermatofibromas under a group by pointing out that they had several clinical-pathological variances [7]. In the study of Horenstein et al. [8] evaluating 484 dermatofibroma cases in 2000, by defining the “eroded” variances, in which the epidermis had partially or totally disappeared, and the “ulcerative” variances with deeper destruction, the authors had emphasized that these lesions should be included in this group. Since superficial changes such as erosion and ulceration are rarely seen in dermatofibromas, there is a small amount of literature data on this subject [9].

It is difficult to make the differential diagnosis between dermatofibrosarcoma protuberans and ulcerative dermatofibroma, since the size of the lesion is bigger than the classical dermatofibroma, with the localization being in locations other than the lower extremities; furthermore, the histopathological presence of superficial destruction, the intense cellular appearance, the swirl-like distribution (storiform), and the observation of penetration to the deep dermis and subcutaneous tissue make the differential diagnosis difficult [8–10]. Immune-histochemical studies performed with the use of Factor XIIIa and CD34 antibodies may be helpful in such cases [4, 5]. A positive staining of CD34 and a negative staining of Factor XIIIa are observed in dermatofibrosarcoma protuberans; a negative staining of CD34 and a positive staining of Factor XIIIa are observed in dermatofibroma [5, 8].

Although this 10 × 8 cm sized tumoral lesion case had a vegetative appearance and clinical characteristics similar to dermatofibrosarcoma protuberans, it was accepted as ulcerative dermatofibroma due to its histopathological appearance of not having atypical cells and mitotic activity, while staining negative with CD34. Consequently, ulcerative dermatofibroma, which is seen rarely and which leads to difficulties in clinical and histopathological diagnosis, should be kept in mind in the differential diagnosis of dermatofibrosarcoma protuberans.
